# Effect of volume expansion with hypertonic- and isotonic saline and isotonic glucose on sodium and water transport in the principal cells in the kidney

**DOI:** 10.1186/1471-2369-14-202

**Published:** 2013-09-26

**Authors:** Janni M Jensen, Frank H Mose, Jesper N Bech, Soren Nielsen, Erling B Pedersen

**Affiliations:** 1 Department of Medical Research, Holstebro Hospital, Laegaardvej 12, Holstebro 7500, Denmark; 2 Aarhus University, Aarhus, Denmark; 3 Water and Salt Research Centre, Aarhus University, Aarhus, Denmark

**Keywords:** Healthy subjects, Urination, Aquaporin2, Epithelial sodium channels, Arginine vasopressin, Renin-angiotensin-aldosterone system

## Abstract

**Background:**

The renal distal nephron plays an important role in the maintenance of sodium balance, extra cellular volume and blood pressure. The degree of water transport, via aquaporin2 water channels (AQP2), and sodium transport, via epithelial sodium channels (ENaC) in renal collecting duct principal cells are reflected by the level of urinary excretion of AQP2 (u-AQP2) and the γ-fraction of ENaC (u-ENaCγ). The effects of an acute intravenous volume load with isotonic saline, hypertonic saline and glucose on u-AQP2, u-ENaCγ and underlying mechanisms have never been studied in a randomized, placebo-controlled trial in healthy humans.

**Methods:**

We studied the effects of 0.9% saline (23 ml/kg), 3% saline (7 ml/kg) and 5% glucose (23 ml/kg) on u-AQP2 and u-ENaCγ, fractional sodium excretion (FE_Na_), free water clearance (C_H2O_), and plasma concentrations of vasopressin (AVP), renin (PRC), angiotensin II (ANG II) and aldosterone (Aldo) in a randomized, crossover study of 23 healthy subjects, who consumed a standardized diet, regarding calories, sodium and fluid for 4 days before each examination day.

**Results:**

After isotonic saline infusion, u-AQP2 increased (27%). C_H2O_ and u-ENaCγ were unchanged, whereas FE_Na_ increased (123%). After hypertonic saline infusion, there was an increase in u-AQP2 (25%), u-ENaCγ (19%) and FE_Na_ (96%), whereas C_H2O_ decreased (-153%). After isotonic glucose infusion, there was a decrease in u-AQP2 (-16%), ENaCγ (-10%) and FE_Na_ (-44%) whereas C_H2O_ increased (164%). AVP remained unchanged after isotonic saline and glucose, but increased after hypertonic saline (139%). PRC, AngII and p-Aldo decreased after isotonic and hypertonic saline infusion, but not after glucose infusion.

**Conclusions:**

Volume expansion with 3% and 0.9% saline increased u-AQP2, while isotonic glucose decreased u-AQP2. Infusion of hypertonic saline increased u-ENaCγ, whereas u-ENaCγ was not significantly changed after isotonic saline and tended to decrease after glucose. Thus, the transport of water and sodium is changed both via the aquaporin 2 water channels and the epithelial sodium channels during all three types of volume expansion to regulate and maintain water- and sodium homeostasis in the body.

**Trial registration:**

Clinical Trial no: NCT01414088

## Background

The distal nephron plays an important role in the maintenance of sodium balance, extra cellular fluid volume and blood pressure [1]. It is well known that inappropriate water and sodium retention is thought to be a key factor in several forms of hypertension, and that aquaporin2 waterchannels (AQP2) play a key role in several water balance disorders [2,3]. Animal models have shown a reduced AQP2 expression in conditions with acquired nephrogenic diabetes insipidus such as lithium treatment and an increased expression in diseases with water retention such as congestive heart failure [4]. Gain-of-function mutations in the epithelial sodium channels (ENaC) cause inappropriate renal sodium retention and consequent increases blood pressure [5]. Because AQP2 and ENaC plays such an important role in water and sodium balance and associated disorders, it is important to identify factors involved in the reabsorption of water and sodium by the kidneys in order to study these disorders in the future.

The exact role of AQP2 and ENaC has never been examined under volume expansion in healthy humans. The effect of an acute intravenous volume load, with isotonic and hypertonic saline and glucose, on urinary excretion of AQP2 (u-AQP2), urinary excretion of ENaC gamma subunit fractions (u-ENaCγ) and its relationship to vasopressin (AVP) and kidney function in healthy humans has not been studied by simultaneous measurement of other important regulatory hormones of water and sodium homeostasis such as the renin-angiotensin-aldosterone system (RAAS).

In this present study we wanted to study the sodium and water transport in the distal nephron by measuring 1) the excretion of u-AQP2 and u-ENaCγ 2) associated regulating hormones and 3) the renal response after volume expansion in healthy humans.

In order to analyse these physiological mechanisms, we performed a randomized, crossover study in healthy subjects. We investigated the effects of infusion with isotonic- and hypertonic saline and isotonic glucose on urinary excretion of AQP2 and ENaCγ corrected for creatinine (u-AQP2_CR_ and u-ENaCγ_CR_), renal function and sodium handling, vasoactive hormones and systemic blood pressure.

## Methods

### Participants

#### ***Inclusion criteria***

Healthy non-smoking men and women with age between 18 – 45 years were included in this study.

#### ***Exclusion criteria***

Subjects with clinical signs or history of heart, lung, kidney, endocrine or malignant disease; abnormal findings in ECG, urine dipstick or biochemistry (blood cell count, plasma concentrations of haemoglobin, sodium, potassium, creatinine, albumin, glucose, bilirubin, alanine aminotransferase, alkaline phosphatase and cholesterol); arterial hypertension (ambulatory BP >130/80 mmHg); medical treatment; alcohol and substance abuse; present smoking; pregnancy; breast feeding; donation of blood within one month prior to the study and obesity (BMI > 32 kg/m^2^) were excluded from this study.

#### ***Withdrawal criteria***

Subjects who developed the condition given in exclusion criteria during the course of the experiment, who withdrew their informed consent, and who had a poor compliance were withdrawn from this study.

### Ethics

This study was approved by the Regional Committee on Health Research Ethics (j. no. M-2011003) and carried out in accordance with the Helsinki Declaration. Written informed consent was obtained from all subjects.

### Recruitment

Healthy male and female volunteers were recruited through advertisement at public institutions in Holstebro, Denmark.

### Design

The study was conducted as a randomized, placebo-controlled, crossover study. On three different occasions separated by at least two weeks, subjects were randomised to 0.9% isotonic saline (0.9% NaCl), 3.0% hypertonic saline (3% NaCl) or 5% glucose (glucose), which was administered as a sustained infusion over 60 minutes.

The amount of fluid used in this study was 0.9% NaCl: 23 ml/kg, 3% NaCl: 7 ml/kg and glucose: 23 ml/kg.

### Effect variables

The main effect variables were u-AQP2, as well as u-ENaCγ. Other effect variables were diastolic (DBP) and systolic blood pressure (SBP), plasma concentration of renin (PRC), angiotensin II (AngII), aldosterone (Aldo), vasopressin (AVP), free water clearance (C_H2O_)_,_ glomerular filtration rate (GFR), fractional excretion of sodium (FE_Na_) and potassium (FE_K_), plasma sodium (p-Na), urine osmolality (u-osm), plasma osmolality (p-osm) and plasma albumin (p-alb).

### Number of subjects

Using a significance level of 5% and a power of 90% it was calculated that the number of subjects needed were 21, when the minimal relevant difference in U-ENaCγ was 100 ng./min and SD was 95 ng./min. Incomplete voiding during examination days was expected in some subjects, therefore 26 subjects were included in the study.

### Experimental procedures

#### ***Experimental procedure prior to the study day***

Four days prior to each study day, subjects consumed a standardized diet regarding calories, sodium and fluid. The diet consisted of 11,000 kJ/day with an energy distribution of 55% carbohydrates, 30% fat and 15% protein in accordance to general dietary guidelines. The sodium content was 120 mmol pr. day. The subjects were asked to drink exactly 2500 ml/day. No alcohol, coffee, tea or soft drink consumption was allowed while on the standardized diet. Subjects were instructed to keep their physical activity as usual during the experiments and to abstain from hard training.

A 24-hour urine collection, ending at 7:00 AM on the examination-day, was used to assess water and sodium balance.

#### ***Experimental procedure on the study day***

After an overnight fast, subjects arrived at our facility at 8:00 AM. Two indwelling catheters for blood sampling and administration of ^51^Cr –EDTA and fluid were placed in both cubital veins. Every 30 minutes, starting at arrival, participants received a 175 ml oral water load of tap water. Urine was collected in standing or sitting position. Otherwise subjects were kept in supine position in a quiet temperature-controlled room (22-25°C).

Three 30 minutes baseline clearance periods were obtained from 9:30 AM to 11:00 AM. These were followed by one clearance period from 11:00 AM to 12:00 PM during which a sustained infusion of either glucose, 0.9% NaCl or 3% NaCl was administered. The post infusion period consisted of three 30-minute periods from 12:00 PM to 1:30 PM. Blood and urine samples were collected every 30 minutes from 9:30 AM to 1:30 PM and analysed for ^51^Cr-EDTA, electrolytes and osmolality. Analysis of plasma concentrations of PRC, Ang II, Aldo and AVP were conducted from blood samples drawn at 11:00 AM (baseline), 12:00 AM (cessation of fluid infusion), 12:30 PM (30 min after cessation of fluid) and 1:30 PM (90 min after cessation of fluid). For data analysis, the 30-minute periods were subdivided into: baseline (0–90 min), infusion (90–150 min) and post infusion (150–180 min, 180–210 min, 210–240 min).

### Measurements

#### ***Renal function***

Glomerular filtration rate was measured by the constant infusion clearance technique using ^51^Cr-EDTA as reference substance. A priming dose of ^51^Cr-EDTA was given followed by sustained infusion that was kept stable using a volume-controlled infusion pump. More than 15% variation in GFR between the three baseline periods led to exclusion of analysis.

#### ***Blood samples***

Were centrifuged for 10 minutes at 2200 × g at 4°C. Plasma hormone samples were kept frozen at -20°C (AngII) and -80°C (PRC, Aldo, and AVP) until assayed. Renin in plasma was determined using an immunoradiometric assay from and a kit from CIS Bio International, Gif-Sur-Yvette Cedex, France. Minimal detection level was 1 pg./mL the coefficients of variation were 14.5% (interassay) and 4.5% (intra assay). Aldosterone in plasma was determined by radioimmunoassay using a kit from Demeditec Diagnostics Systems Laboratories Inc. (Webster, TX, USA). Minimal detection level was 22 pmol/L. The coefficients of variation were 8.2% (inter-assay) and 3.9% (intra-assay). Arginine vasopressin and Angiotensin II were extracted from plasma with C_18_ Sep-Pak (water associates, Milford, MA, USA) and subsequently measured using radioimmunoassay as previously described. The antibody against angiotensin II was obtained from the Department of Clinical Physiology, Glostrup Hospital, Glostrup, Denmark. Minimal detection level was 2 pmol/L. The coefficients of variation were 12% (inter-assay) and 8% (intra-assay). The antibody against AVP was a gift from Professor Jacques Dürr (Miami, FL, USA). Minimal detection level was 0.2 pmol/L. The coefficients of variation were 13% (inter-assay) and 9% (intra –assay).

#### ***Urine samples***

Were kept frozen at -20°C until assayed. U-AQP2 was measured by radioimmunoassay as previously described [6,7]. Antibodies were raised in rabbits to a synthetic peptide corresponding to the 15 COOH-terminal amino acids in human AQP2 to which was added an NH_2_-terminal cystein for conjugation and affinity purification. Minimal detection level was 34 pg/tube/tube. The coefficients of variation were 11.7% (inter-assay) and 5.9% (intra-assay). U-ENaCγ was measured by radioimmunoassay as previously described [8,9]. Antibodies were raised against the synthetic ENaCγ peptide in rabbits and affinity purified as described previously [10]. Minimal detection level was 48 pg/tube. The coefficients of variation were 14% (inter-assay) and 6.7% (intra-assay).

#### ***Blood pressure measurement***

Brachial blood pressure was recorded using a semiautomatic oscillometric devise (Omron 705IT, Omron Matsusaka, Japan).

Plasma and urine concentrations of sodium, potassium, creatinine and albumin were measured using routine methods at the Department of Clinical Biochemistry, Holstebro Hospital.

Fractional excretion of sodium was calculated as [sodium clearance (*C*_Na_)/GFR × 100%].

Fractional excretion of potassium was calculated as [potassium clearance (C_K_)/GFR × 100%].

Free water clearance was calculated as [urine output (UO) – osmolar clearance (*C*_OMS_)].

*C*_OSM_ was calculated as [urine osmolarity/plasma osmolarity × UO].

### Statistics

Statistical analyses were performed using IBM SPSS statistics version 20.0.0 (IBM Corp.; Armonk, NY, USA). Single baseline values were obtained by taking the weighed average of the measurements from the three baseline periods. Parametric data are presented as means ± standard deviation (SD) and nonparametric data as medians with interquartile ranges. General linear model (GLM) with repeated measures was performed, with time as within-subject factor and intervention as between subject factor, to test for differences within and between groups. One-way ANOVA was used for comparison of means between groups when differences were found. For non-parametric data related samples Friedman’s two-way analysis (FM) was used. For comparison within groups at baseline and post infusion period 210–240 minutes, a paired t-test was used when data were parametric and Wilcoxon’s signed rank test was used when data were nonparametric. Statistical significance was defined as p < 0.05 in all analyses.

## Results

### Demographics

A total of 31 healthy women and men were enrolled in the study. Five subjects were excluded due to: abnormal blood samples (1), 24-h BP above 130/80 mmHg (1), non-compliance (1) and withdrawal of informed consent (2). Thus, 26 persons completed the study. Three were not able to void satisfactorily during clearance experiments and were excluded from analysis. One was not able to void in two post intervention periods after 3% NaCl and was excluded in channel analysis only.

The remaining 23 males (n = 9) and women (n = 14) had a median age of 26 years (range 18–42) and a mean BMI of 24.4 ± 2.3 kg/m^2^. Mean ambulatory blood pressure was 119/70 ± 8/4 mmHg. Screening blood values were b-haemoglobin 8.5 ± 0.7 mmol/L, p-sodium 139 ± 2, p-potassium 3.9 ± 0.4 mmol/L, p-creatinine 74 ± 9 μmol/L, p-albumin 42 ± 3 g/L, p-glucose 5.1 ± 0.6 mmol/L, p-alanine transaminase 25 ± 9 U/L and p-cholesterol 4.5 ± 0.5 mmol/L.

### Twenty-four-hour urine collection

Table 1 shows the results of the 24-h urine collection in 23 healthy subjects after 4 days of standardized diet. Mean u-AQP2, u-ENaCγ, urinary sodium, urine osmolarity, C_H2O_ and urine volume were the same in all three examination days indicating that the subjects had kept their supplied diets and fluid intake.

**Table 1:** **Urine output, urine osmolarity (u-osm), free water clearance (C**_**H2O**_**), urinary AQP2 excretion per minute (u-AQP2), urinary excretion of ENaCγ per minute (u-ENaCγ), urinary sodium excretion (u-Na) and fractional excretion of sodium (FE**_**Na**_**) during 24-hours urine collection with fluid deprivation (12 PM to 8.00 AM) in a randomised, crossover study of 23 healthy subjects**

	**Examination day:**			**P (ANOVA)**
	**0.9% NaCl**	**3% NaCl**	**Glucose**	
**Urine Output (ml/24 h)**	2306(559)	2120(695)	2281(650)	0.56
**u-osm (mosm/24 h)**	848(231)	797(207)	833(239)	0.73
**C**_**H2O **_**(ml/min)**	-0.46(0–61)	-0.47(0.57)	-0.44(0.64)	0.99
**u-AQP2 (ng/min)**	0.93(0.24)	0.91(0.22)	0.93(0.24)	0.89
**u-ENaCγ (pg/min)**	405(163)	389(188)	385(164)	0.92
**u-Na (mmol/24 h)**	124(52)	119(41)	120(56)	0.93
**FE**_**Na **_**(%)**	0.49(0.15)	0.47(0.13)	0.45(0.18)	0.67

Values are means with SD in brackets. One-way ANOVA was used for comparison between groups.

### Water excretion, u-AQP2, u-osm

Table 2 shows the absolute values of UO, C_H2O_, u-AQP2_CR,_ u-AQP2 excretion rate and u-osm during the baseline period, the infusion period and the post infusion period.

**Table 2:** **Effect of 0.9% isotonic saline (0.9% NaCl), 3% hypertonic saline (3% NaCl) and isotonic glucose (Glucose) on urinary output (OU), free water clearence (C**_**H2O**_**), urine osmolarity (u-osm), urinary aquaporin2 excretion rate (u-AQP2) and urinary aquaporin2 corrected for creatinine (u-AQP2**_**CR**_**) in a randomized, crossover study of 23 healthy subjects**

**Periods**	**Baseline**	**Infusion**	**Post infusion**			**p (GLM-within)**
	**0-90 min**	**90-150 min**	**150-180 min**	**180-210 min**	**210-240 min**	
**UO (ml/min)**						
0.9% NaCl	6.90(1.33)	8.75(1.76)	7.95(2.31)	7.39(2.36)	8.12(2.26)	<0.0001
3% NaCl	7.12(1.35)	2.75(0.73)	1.81(0.64)	1.98(1.07)	2.05(1.09)**	
Glucose	6.83(1.58)	11.84(2.28)	16.05(2.82)	15.28(2.47)	13.45(2.47)**	
p (GLM between)		<0.0001				
p ANOVA	0.77	<0.0001	<0.0001	<0.0001	<0.0001	
**C**_**H2O**_						
0.9% NaCl	3.87(1.55)	5.29(1.44)	4.07(2.09)	3.01(2.04)	3.62(1.59)	<0.0001
3% NaCl	3.97(1.21)	-0.35(0.73)	-1.87(0.83)	-2.22(0.88)	-2.26(0.96)	
Glucose	3.84(1.32)	6.38(1.77)	11.60(0.52)	12.47(2.35)	11.17(2.12)	
p (GLM between)		<0.0001				
p ANOVA	0.95	<0.0001	<0.0001	<0.0001	<0.0001	
**U- osm (mosmol/kg)**						
0.9% NaCl	140(62)	115(21)	149(58)	176(50)	161(33)	<0.0001
3% NaCl	136(37)	342(96)	606(114)	637(105)	640(111)*	
Glucose	136(41)	133(21)	79(21)	52(12)	48(7)*	
p (GLM between)		p <0.0001				
p ANOVA	0.95	<0.0001	<0.0001	<0.0001	<0.0001	
**u-AQP2 (ng/min)**						
0.9% NaCl	1.03 (0.25)	1.02(0.22)	1.22(0.34)	-	1.26(0.32)*	<0.0001
3% NaCl	1.02(0.22)	0.99(0.31)	1.28(0.30)	-	1.37(0.34)*	
Glucose	1.01(0.22)	1.26(0.25)	1.19(0.29)	-	0.78(0.18)*	
p (GLM between)		0.274				
**u-AQP2**_**CR **_**(ng/mmol)**						
0.9% NaCl	102.5(19.9)	110.8(18.1)	119.8(25.4)	-	125.0(21.0)**	<0.0001
3% NaCl	105.9(15.7)	108.6(27.2)	125.1(31.4)	-	132.7(26.5)*	
Glucose	105.7(19.1)	137.1(22.3)	118.1(26.5)	-	87.9(15.4)*	
p (GLM between)		0.565				

Values are mean with SD in brackets. General linear model (GLM) with repeated measures was performed for comparison within the group and intervention as between subjects factor. One way ANOVA was performed when differences were found between interventions. Paired t-test was used for comparison within treatment group at baseline and post infusion period 210 – 240 minutes. *p < 0.0001; **p < 0.001.

UO increased significantly after 0.9% NaCl and glucose. The 3% NaCl infusion induced a significantly and sustained decrease in UO. The relative changes in UO were significantly different between the three interventions.

C_H2O_ increased during the infusion with 0.9% NaCl, and decreased slightly, although significant in the postinfusion period. At the end of the examination-day C_H2O_ increased towards baseline levels with an over all relative change of -10%. There was a pronounced increase in C_H2O_ after glucose, whereas C_H2O_ decreased after 3% NaCl and changed from positive values at baseline to negative values after infusion. Thus, indicating a change from free water excretion to water reabsorption (Table 2).

**Figure 1:**
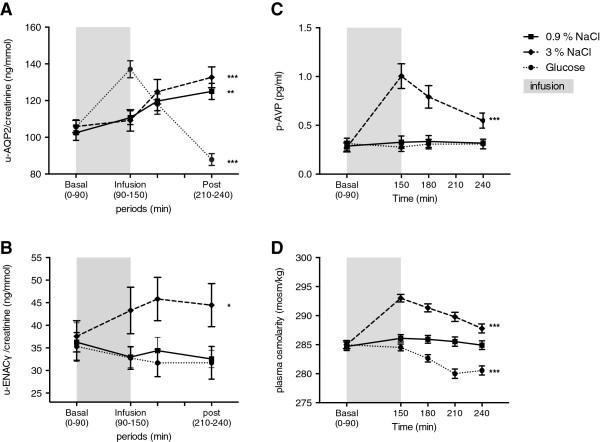
**Effects of isotonic 0.9% saline (■), hypertonic 3% saline (**♦**) and isotonic glucose (**●**) on urinary excretion of A) u-AQP2 and B) u-ENaCγ adjusted to creatinine, C) plasma concentration of vasopressin (AVP) and D) plasma osmolality in 23 healthy subjects.** Values are means ± SEM. Paired t-test was used for comparison of post infusion period 210–240 min vs. baseline. * p < 0.01; ** p < 0.001; *** p < 0.0001.

U-AQP2_CR_ increased by 27% (p < 0.001) in response to 0.9% and by 26% (p < 0.0001) after 3% NaCl and reached maximum at 240 min after baseline. During glucose infusion (90–150 min) there was a primary increase in u-AQP2_CR_ after which u-AQP2_CR_ decreased and reached a minimum of - 16% (p < 0.0001) at 210–240 min (Figure 1A). The excretion of u-AQP2 divided by gender, showed that u-AQP2_CR_ tended to be higher in women than in men, but there was no statistical significant difference. This was due to a lower creatinine concentration in women’s urine (data not shown). U-AQP2 excretion rate followed the same pattern (Table 2). The relative changes in u-AQP2 did not differ between 3% NaCl and 0.9% NaCl, but both were significantly different from the relative change in u-AQP2 after glucose infusion.

U-Osm decreased during 0.9% NaCl infusion with minimum after infusion ended at 150 minutes, after which u-osm increased, coherent with the changes seen in C_H2O_. U-osm increased significantly in response to 3% NaCl and lasted throughout the experiment. During glucose infusion u-osm remained constant for 60 minutes until glucose infusion was completed, after which u-osm declined and reached minimum at 210–240 min (Table 3).

**Table 3:** **Effect of 0.9% isotonic saline (0.9% NaCl), 3% hypertonic saline (3% NaCl) and isotonic glucose (Glucose) on urinary sodium excretion (u-Na), fractional excretion of sodium (FE**_**Na**_**), urinary potassium wxcretion (u-K), fractional excretion of potassium (FE**_**K**_**), urinary gamma fraction of the epithelial sodium channels excretion rate (u-ENaCγ) and urinary gamma fraction of ENaC corrected for creatinine (u-ENaCγ**_**CR**_**)**

**Periods**	**Baseline**	**Infusion**	**Post infusion**			**p (GLM within)**
	**0-90 min**	**90-150 min**	**150-180 min**	**180-210 min**	**210-240 min**	
**u-Na (mmol/min)**						
0.9% NaCl	1.24(0.55)	1.83(0.56)	2.51(1.05)	2.78(0.99)	3.07(0.98)*	<0.0001
3% NaCl	1.38(0.58)	1.86(0.75)	2.65(1.06)	2.94(1.44)	3.17(1.38)*	
Glucose	1.23(0.51)	1.38(7.3)	0.94(0.43)	0.83(0.35)	0.72(0.35)*	
p (GLM between)		<0.0001				
p (ANOVA)	0.599	0.028	<0.0001	<0.0001	<0.001	
**FE**_**Na**_						
0.9% NaCl	1.26(0.53)	1.93(0.61)	2.35(0.72)	2.67(0.76)	2.80(0.75)*	<0.0001
3% NaCl	1.44(0.61)	2.08(0.81)	2.66(1.07)	3.07(1.27)	3.01(1.26)*	
Glucose	1.27(0.53)	1.36(0.64)	0.88(0.37)	0.81(0.37)	0.74(0.37)*	
p (GLM between)		<0.0001				
p (ANOVA)	0.465	0.002	<0.0001	<0.0001	<0.0001	
**U-K (mmol/min)**						
0.9% NaCl	26.5(9.1)	22.5(7.6)	21.1(8.9)	22.7(12.2)	23.5(10.2)***	<0.0001
3% NaCl	25.4(9.2)	17.3(7.3)	16.9(8.1)	20.1(10.5)	21.1(9.9)***	
Glucose	24.9(8.7)	17.2(5.8)	10.8(3.7)	9.6(3.2)	11.23(4.4)*	
p (GLM between)		<0.0001				
p (ANOVA)	0.827	0.016	<0.0001	<0.0001	<0.0001	
**FE**_**K**_						
0.9% NaCl	28.2(9.9)	23.3(7.4)	20.4(7.8)	21.5(8.2)	21.5(7.9)***	<0.0001
3% NaCl	26.3(9.0)	18.5(6.8)	17.0(7.5)	20.9(9.3)	20.0(8.9)***	
Glucose	25.7(8.8)	16.7(5.5)	9.8(3.1)	9.3(3.4)	11.5(4.4)*	
p (GLM between)		< 0.0001				
p (ANOVA)	0.624	<0.01	<0.0001	<0.0001	<0.0001	
**u-ENaCγ (ng/min)**						
0.9% NaCl	351.2(155.0)	302.3(108.5)	341.2(123.0)	-	327.2(111.3)	<0.0001
3% NaCl	358.8(160.0)	375.9(173.7)	462.6(203.4)	-	435.9(168.7)**	
Glucose	338.3(150.0)	301.7(128.8)	335.2(185.1)	-	283.7(164.4)***	
p (GLM between)		0.076				
**u-ENaCγ**_**CR **_**(ng/mmol)**						
0.9% NaCl	36.25(3.63)	32.98(3.50)	34.40(3.63)	-	32.56(3.57)	<0.001
3% NaCl	37.56(3.70)	43.29(3.58)	45.83(3.72)	-	44.46(3.65)***	
Glucose	35.33(3.62)	32.78(3.50)	31.68(3.63)	-	31.71(3.57)	
p (GLM between)		0.091				

Values are means with SD in brackets. General linear model (GLM) with repeated measures was performed for comparison within the group and intervention as between subjects factor. One way ANOVA was used for comparison of means between subjects when differences were found. Paired t-test was used for comparison within treatment group at baseline and third post infusion period *p < 0.0001, **p <0.001, *** p < 0.01.

### Sodium excretion, u-ENaCγ, u-Na, FE_Na_, u-K and FE_K_

Table 3 shows the absolute values of u-Na, FE_Na_, u-K, FE_K_, u-ENaCγ_CR_ and u-ENaCγ excretion rate during the baseline period, the infusion period and the post infusion period.

Infusion with 0.9% NaCl and 3% NaCl were accompanied by a significant and similar increase in u-Na and FE_Na_ that lasted throughout the experiment. There were no significant differences between 0.9% NaCl and 3% NaCl infusions. In contrast, U-Na and FE_Na_ decreased after glucose infusion. The relative changes in u-Na and FE_Na_ were significant lower after glucose compared to both saline infusions.

U-K and FE_K_ decreased significantly after all three infusions, but with the greatest extend after glucose infusion. In the post infusion period (150–240 min) the excretion of potassium in urine increased slightly more after 3.0% NaCl than 0.9% NaCl, but did not reach baseline levels.

U-ENaCγ_CR_ decreased slightly, but non-significantly during 0.9% NaCl and glucose infusions. A significant increase was seen in u-ENaCγ_CR_ in response to 3% NaCl (p < 0.01) (Figure 1B) and the relative increase in u-ENaCγ_CR_ were significantly higher in response to 3% NaCl compared to 0.9% NaCl and glucose. Divided by gender the differences in u-ENaCγ_CR_ showed no statistical significant difference, although u-ENaCγ_CR_ tended to be higher in women due to the lower urine creatinine (data not shown). U-ENaCγ excretion rate followed the same pattern with regard to saline infusions, whereas a significantly lower u-ENaCγ excretion rate occurred after glucose infusion (Table 3).

### Vasoactive hormones

**Figure 2:**
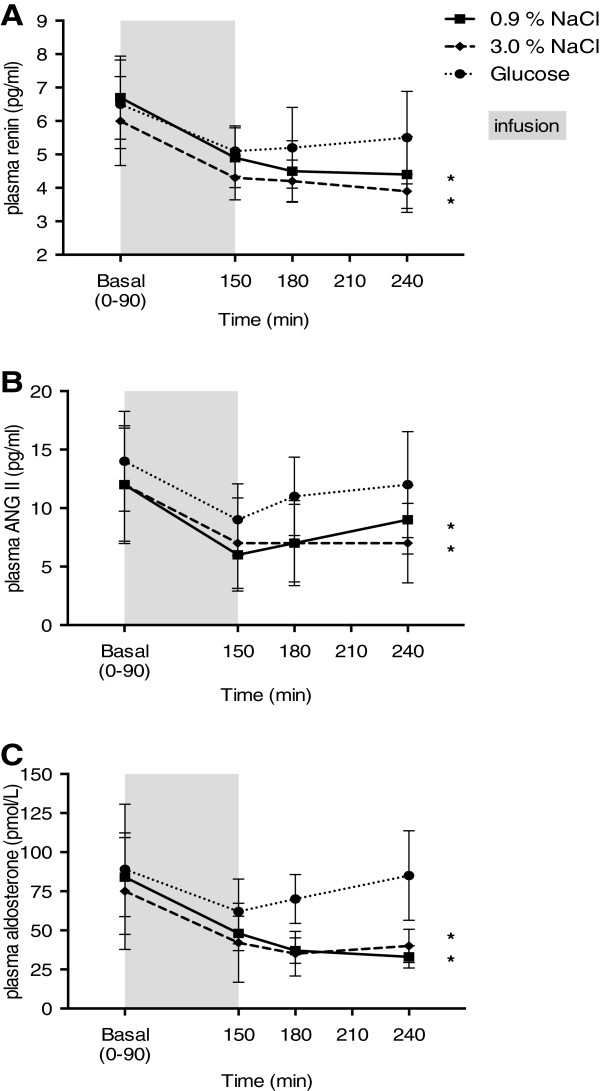
**Effects of isotonic 0.9% saline (■), hypertonic 3% saline (**♦**) and isotonic glucose (**●**) on plasma renin (A), plasma angiontensin II (B) and plasma aldosterone (C) concentrations.** Values are expressed as mean ± SEM. General linear model (GLM) with repeated measures within subjects was significant for all three variables. Paired t-test was used for comparison within treatment groups at postinfusion 240 min vs. basal. * p < 0.0001.

PRC, Ang II and Aldo were suppressed to the same extent in all three parameters in response to 0.9% NaCl and 3% NaCl with no significant difference between interventions. There was a primary decrease during glucose infusion (90–150 min), but when infusion ceased values returned to baseline levels with no overall significant change (Figure 2).

AVP did not change in response to 0.9% NaCl and glucose, but increased significantly after 3% NaCl with a maximum at 150 minutes and a steady fall during the post infusion period (Figure 1C).

### Blood pressure, pulse rate, GFR, p-Na, p-alb and p-osm

Table 4 shows the absolute values of systolic and diastolic blood pressure, pulse rate, GFR, plasma sodium and plasma albumin during the baseline period, the infusion period and the post infusion period.

**Table 4:** **Effect of 0.9% isotonic saline (0.9% NaCl), 3.0% hypertonic saline (3% NaCl) and 5% glucose (Glucose) on **^**51**^**Cr-EDTA-clearance, plasma sodium, plasma albumin, systolic blood pressure (SBP), diastolic blood pressure (DBP) and pulse rate in a randomized, placebo-controlled, crossover study of 23 healthy subjects**

**Periods**	**Baseline**	**Infusion**	**Post infusion**			**p (GLM-within)**
	**0-90 min**	**90-150 min**	**150-180 min**	**180-210 min**	**210-240 min**	
^**51**^**Cr-EDTA-clearance (ml/min/1.73 m**^**2**^**)**						
0.9% NaCl	98.1(10.1)	97.2(9.9)	100.0(12.4)	106.4(19.5)	106.1(13.3)**	0.002
3% NaCl	95.7(10.4)	93.0(11.4)	96.6(14.8)	97.0(14.8)	101.8(13.9)***	
Glucose	96.9(10.3)	100.0 (9.9)	105.9(10.5)	104.0(13.9)	99.1(9.7)	
p (GLM between)		0.244				
**p-Sodium (mmol/l)**						
0.9% NaCl	138.1(2.1)	139.4(2.1)	139.6(2.4)	139.1(2.4)	138.7(2.3)**	<0.0001
3% NaCl	138.0(2.0)	142.5(1.8)	141.7(1.7)	141.1(2.1)	139.7(2.0)*	
Glucose	138.1(2.0)	128.7(3.7)	134.1(3.6)	135.7(3.7)	136.9(2.9)*	
p (GLM between)		<0.0001				
p (ANOVA)	0.94	<0.0001	<0.0001	<0.0001	=0.001	
**p-albumin (g/L)**						
0.9% NaCl	40.3(2.6)	40.1(2.4)	35.3(2.0)	35.8(2.1)	36.0(2.2)*	<0.0001
3% NaCl	40.1(2.6)	39.8(2.6)	35.2(2.2)	36.0(2.3)	36.2(2.7)*	
Glucose	40.9(2.2)	40.3(2.8)	36.1(2.5)	39.1(2.7)	39.7(2.9)*	
p (GLM between)		<0.05				
p (ANOVA)	0.510	0.765	0.328	<0.0001	<0.0001	
**SBP (mmHg)**						
0.9% NaCl	114.5(10.6)	117.7(10.4)	116.6(11.0)	116.9(11.6)	117.7(11.5)	0.439
3% NaCl	114.0(9.5)	117.9(9.1)	117.2(9.2)	115.5(10.0)	116.4(9.7)	
Glucose	115.5(8.4)	118.2(9.8)	117.7(9.1)	115.5(8.2)	117.0(9.1)	
p (GLM between)		0.975				
**DBP (mmHg)**						
0.9% NaCl	62.9(4.3)	64.0(6.1)	62.2(5.7)	63.3(5.6)	63.4(5.1)	0.038
3% NaCl	62.9(4.2)	62.1(5.0)	61.0(5.4)	61.4(4.9)	62.9(4.9)	
Glucose	64.4(4.2)	65.8(5.6)	65.3(6.3)	65.5(5.3)	63.6(7.5)	
p (GLM between)		0.12				
**Pulse rate**						
0.9% NaCl	54.1(11.0)	57.2(11.9)	56.4(11.6)	58.0(12.1)	57.4(11.9)*	<0.0001
3% NaCl	54.0(10.5)	58.2(11.2)	57.0(11.2)	56.9(11.3)	57.7(11.5)*	
Glucose	54.7(10.5)	57.4(12.5)	57.1(12.0)	60.3(11.7)	61.3(12.3)*	
p (GLM between)		0.917				

Values are mean with SD in brackets. General linear model (GLM) with repeated measures was performed for comparison within the group and intervention as between subjects factor. One way ANOVA was performed for comparison of means when differences were found between intervensions. Paired t-test was used for comparison within treatment groups at baseline and post infusion period 210 – 240 minutes. *p < 0.0001; **p < 0.001; ***p < 0.05.

Systolic BP was the same after all three infusions. There was a small difference in diastolic BP pattern during the examination day, but the changes were very small and might be by chance. During the examination day pulse rate increased slightly in response to 0.9% NaCl and 3% NaCl, while the heart rate increased to a higher extent in response to the glucose infusion (Table 4). The increase in pulse rate did not differ between 0.9% saline and 3% saline, but there was a difference in the relative increase in pulse rate between saline and glucose infusion (p < 0.01).

GFR increased slightly, although significantly, on the examination day. However the changes were very small (Table 4).

P-Na increased in response to both 0.9% NaCl and 3% NaCl with maximum after 150 minutes. In response to glucose p-Na decreased markedly after 150 minutes to a mean of 128.7 mmol/l (Table 4). The increase was higher after 3% NaCl compared to 0.9% NaCl and accordingly the changes after glucose were lower compared to saline.

P-alb decreased significantly in response to 0.9%, 3% NaCl and glucose infusions. The decline was significantly lower and sustained after both saline infusions compared to glucose, which is related to an expected increase in extracellular fluid.

P-osm increased slightly during 0.9% NaCl infusion, but remained unchanged at the end of the examination day. P-osm increased significantly in response to 3% NaCl, with a maximum of 293 mosm/kg and decreased significantly after glucose to 280 mosm/kg at 150 min. The changes in p-osm indicated that isotonic, hypertonic and hypotonic conditions were established (Figure 1D).

### Fluid, sodium balance and body weight during the examination days

The average fluid administered intravenous was 1749 ml of 0.9% NaCl (SD 270), 555 ml of 3% NaCl (SD 90) and 1736 ml of glucose (SD 282). The cumulative water input was 3674 ml (SD 270), 2480 ml (SD 90) and 3661 ml (SD 282) respectively, as participants drank an additional 1925 ml of tap water each examination day. During the examination days the average total urine output was 1858 ml (SD 246) in subjects who received 0.9% NaCl, 984 ml (SD 202) in subjects who received 3% NaCl and 2682 ml (SD 351) in subjects who received glucose. The fraction of water excreted after 240 min was 51% when 0.9% NaCl was infused, 40% when 3% NaCl was infused and 73% when glucose was infused. The total amount of sodium infused was 269 mmol (SD 42) of 0.9% NaCl and 285 mmol (SD 46) of 3.0% NaCl. The cumulative sodium output at 240 min was 50 mmol (SD 16) after 0.9% NaCl, 54 mmol (SD 21) after 3% NaCl and 21 mmol (SD 9) after glucose. The fraction of sodium excreted after 240 min was 19% after both 0.9% and 3% NaCl infusions. This was accompanied by a significant increased bodyweight in response to 0.9% NaCl from 73.2 kg (SD 11.3) at baseline to 74.3 kg (SD 11.4) at the end of the study day [+1.1 kg (SD 0.39); p < 0.0001], in response to 3% NaCl from 73.3 kg (SD 11.6) at baseline to 74.1 kg (SD 11.7) at the end of the study day [+0.8 kg (SD 0.39); p < 0.0001] and to a smaller extent in response to glucose from 72.8 kg (SD 11.8) at baseline to 73.1 kg (SD12.0) at the end of the study day [+0.3 kg (SD 0.5); p <0.05].

## Discussions

In the present study we examined the effect of an acute intravenous volume load of 0.9% saline, 3% saline and isotonic glucose infusions on u-AQP2 and u-ENaCγ in a randomized, crossover study of healthy subjects. The purpose was to evaluate the transport activity via the aquaporin 2 water channels and the epithelial sodium channels in the principal cells in the distal part of the nephron.

During infusion and in the period immediately after, adaptive physiological changes take place in renal function and vasoactive hormones. Thus, the main changes in the effect variables could be expected to occur after the infusion. In the present study, we paid special attention to changes in the effect variables in the last post infusion period (Post infusion 210–240), i.e. 60–90 minutes after infusion had ceased. During this period, u-AQP2 increased after hypertonic and isotonic saline infusion and decreased after glucose infusion. At the same time, u-ENaCγ increased after hypertonic saline infusion and remained unchanged after isotonic saline and glucose infusion.

### U-AQP2 after infusion with hypotonic and isotonic saline and isotonic glucose

Aquaporin-2 (AQP2) is located in the collecting duct principal cells [11] and is expressed in the apical plasma membrane [12]. Vasopressin (AVP) regulates AQP2 by binding to V2 receptors in the basolateral membrane, [11,13]. Short term exposure to AVP causes trafficking and insertion of the intracellular vesicles, containing AQP2, to the apical membrane and increases the water permeability and absorption [11-14]. Long-term regulation occurs over a period of hours to days, and is caused by AVP-regulated gene transcription resulting in an increase in AQP2 whole-cell abundance [4,13]. Experiments in rats showed that infusion of dDAVP increased u-AQP2 [15]. This is consistent with the view that increased delivery of AQP2 channels to the apical membrane results in increased excretion of AQP2 after stimulation with AVP [6,15-21]. Approximately 3% of AQP2 in the collecting duct are excreted into urine [20], but the underlying mechanisms are unknown.

Volume expansion with 3% hypertonic saline increases plasma osmolarity beyond the threshold of the hypothalamic osmoreceptors, triggering release of AVP and a subsequent increase in u-AQP2. Saito et al found a significant relationship between urinary excretion of AQP2 and p-AVP in healthy subjects after 5% hypertonic saline infusion [17]. Pedersen et al found a positive correlation between u-AQP2 and p-AVP during 24 h of water deprivation and after 3% hypertonic saline infusion [6]. Thus, previous studies in humans have demonstrated that the activity of the AQP2 water channels can be determined by measuring u-AQP2 [6-8,17,18]. Surprisingly, Baumgartner et al found no change in u-AQP2 after infusion of 2.5% NaCl in healthy volunteers, despite a significant rise in both urine osmolarity and AVP [22]. However, the oral water load was 3–4 times higher prior to infusion compared to our study. Thus, the large water load before infusion might have overruled the stimulatory effects of hypertonic saline. As expected, our study showed that u-AQP2 increased after 3% NaCl with a corresponding rise in urine osmolarity and a reduction in C_H2O_. Thus, our findings indicate an increased water reabsorption via the aquaporin-2 water channels in the distal tubules. Prior to the increase in u-AQP2, there was an abrupt rise in p-osm and p-AVP induced by the hypertonic saline infusion. Animal studies have shown that hypertonicity can cause an up regulation of AQP2 expression in the apical membrane comparable with that achieved by AVP alone [23,24]. It cannot be excluded that this might play an active part in the increased excretion of u-AQP2. Most likely, the increased water reabsorption was mediated by an increase in p-AVP. U-AQP2 continued to rise throughout the examination day, suggesting that AQP2 channels remained inserted and active in the apical membrane due to actions of elevated p-AVP.

Infusion of isotonic saline depresses the fractional water and salt reabsorption in the proximal tubules in animals [25]. In the present study, infusion with 0.9% NaCl caused the same response in u-AQP2, u-osm and C_H2O_ as 3% NaCl infusion, albeit to a lesser extent. There was a small rise in p-osm to a maximum level of 286 mosmol/kg corresponding to a rise of 0.5%. This increase is below the osmoreceptor threshold, and we did not see, nor expect, any significant change in p-AVP. Therefore AVP could not be the main regulator of AQP2 during 0.9% NaCl. Most likely, the increased water transport via AQP2 is a compensatory phenomenon to antagonize a decrease in the renal water absorption in the proximal tubules, which occurs after isotonic volume expansion. The mechanism might be due to an increased activity in the natriuretic peptide system [26,27].

Infusion of 5% glucose causes a volume expansion distributed throughout the fluid phases in the body with only a very small increase in plasma volume. This is illustrated in the measurements of plasma albumin, where concentrations at 240 min were virtually equal to baseline (Table 4), indicating no change in extracellular fluid. According to our knowledge, no study has measured u-AQP2 after glucose infusion. A study of healthy subjects showed, that after an oral water load of 20 mL/kg for 15 minutes (mean intake 1605 ml) u-AQP2 decreased 17% after 210 minutes [21]. In a recent study, subjects received an oral water load of 20 ml/kg for 15 min (mean intake 1389 ml) with a subsequent 27% decrease in u-AQP2 after 240 minutes [28]. Both plasma osmolarity and p-AVP decreased. Thus, it has been showed that u-AQP2 is reduced during water diuresis after oral water intake [21,28,29].

In our study, subjects received a mean of 1736 ml glucose IV. In the last post infusion period the expected aquaretic response occurred, with a 16% decrease in u-AQP2_cr_, a decrease in u-osm and an increase in UO and C_H2O_. Plasma osmolarity decreased from 285 mosm/kg to 280 m0sm/kg, i.e. a 2% decline, but with no accompanying reduction in p-AVP. Our findings indicate a reduced reabsorption of water via the aquaporin-2 water channels in the distal tubules after isotonic glucose infusion. The lack of change in p-AVP could firstly be explained by the fact that the subjects had received 1225 ml of oral water load prior to the infusion start, and this could have suppressed AVP in baseline periods beforehand. Secondly, the measurements of p-AVP concentration may not be sensitive enough to detect a small decrease. The recent discovered peptide Apelin, may also play a role. Apelin is colozalized with AVP in magnocellular neurons of hypothalamus [30,31]. In healthy male volunteers decreasing plasma osmolarity by waterloading reduced p-AVP modestly but p-Apelin increased rapidly [32]. Apelin regulation is opposite to that of AVP and data suggests that Apelin, like AVP may participate in regulating water homeostasis [32]. We did not measure p-Apelin, but it could have been of interest to investigate plasma apelin in parallel with p-AVP in conditions of different volume expansions.

Thus, in the last post-infusion period, u-AQP2 increased approximately to the same extent after hypertonic and isotonic saline infusions, whereas a marked fall was seen after isotonic glucose infusion. A possible explanation for the delay in changes of u-AQP2 could be that it takes few minutes for changes in AVP to act on the principal cell, either by insertion or removal of AQP2 from the apical membrane, but it takes several minutes before the effect is seen in the excretion of u-AQP2 in the urine.

### U-ENaCγ after infusion with hypotonic and isotonic saline and isotonic glucose

Sodium transport across the collecting duct occurs through the epithelial sodium channel and is responsible for reabsorption of 3–5 % of filtered sodium [33]. ENaC is composed of three distinct subunits: α, β and γ and localized at the apical plasma membrane of principal cells [34,35]. ENaC is a target of aldosterone that acts on the mineralocorticoid receptor. Aldosterone increases sodium transport by redistributing ENaC subunits from intracellular locations to the apical membrane as well as altering gene transcription [33,36,37]. While the action of aldosterone occurs over hours or days, another synergistically pathway involves AVP [35,37-41]. In the cortical collecting ducts in rats, AVP binds to the V2 receptors, stimulates cAMP and increases sodium reabsorption by promoting trafficking and insertion of ENaC into the apical membrane inducing a rapid change in channel activity [34,35,40]. Recent studies in humans demonstrated that AVP, via V2 receptors, stimulates ENaC mediated sodium reabsorption across principal cells [39,42,43].

Fractions of ENaC are normally excreted into the urine. The amount of ENaC-fractions is supposed to reflect the activity of the sodium transport via the epithelial sodium channels just as u-AQP2 reflects the functional status of the AQP2 water channels. Recently, our group introduced a new method to evaluate sodium reabsorption in the principal cells in the distal tubules. Lauridsen et al demonstrated a significant correlation between changes in urinary sodium excretion and changes in urinary excretion of the beta fraction (u-ENaC_β_) in healthy humans [44,45]. Apparently, u-ENaC_β_ can be used as a biomarker for the transport of sodium via ENaC. In the present study, we measured the gamma fraction of the protein of the epithelial sodium channels to evaluate the up-and down regulation of γ-ENaC expression and sodium transport via ENaC as previously reported from our group [9,46].

The sodium-chloride symporter (NCC) in the distal convoluted tubules (DCT) is as another major sodium reabsorbing pathway. Sodium reabsorption in DCT is essential to define the amount of sodium delivery to the principal cells in the collecting duct. It is widely accepted that NCC is regulated by Ang II and aldosterone [47,48]. Studies have also shown that high AVP increase phosphorylation of NCC and presumably result in greater sodium reabsorption [49].

Experimental animal-studies have demonstrated that isotonic and hypertonic saline IV reduced reabsorption of sodium in the proximal tubules, and thereby increased the amount of sodium in the urine [25,50]. Andersen LJ et al studied the effects of hypertonic and isotonic saline in healthy subjects on a controlled diet. The subjects received an IV sodium load of either 25 ml/kg isotonic saline or 4.5 ml/kg 3% hypertonic saline for 90 minutes [51]. Urinary sodium excretion increased in both isotonic and hypertonic saline, with natriuresis after hypertonic saline exceeding that after isotonic saline. Plasma sodium and plasma osmolarity increased substantially after hypertonic saline, as did p-AVP. Our study showed that 3% NaCl infusion increased u-ENaCγ, FE_Na_, p-Osm, p-Na and p-AVP. Thus, our findings reflect an increased sodium reabsorption via ENaC in the principal cells, and furthermore confirmed the results by Andersen et al [51]. The increased u-ENaCγ could partly be explained by a considerable decrease in the renal sodium absorption proximal in the nephron, compensated for and adjusted by an increase in absorption in the distal part. However, the rise in p-AVP seen immediately after 3% NaCl infusion could also indicate that the increased u-ENaCγ is caused by actions of AVP. An increased sodium movement from the lumen to the cell via ENaC would theoretically drive potassium secretion through the ROMK channels [52,53]. Surprisingly we measured a fall in excretion of potassium in the urine. This could argue against a major role of ENaC mediated sodium transport. If NCC increased sodium reabsorption, both to compensate for a decrease in proximal reabsorption and due to high p-AVP, then less sodium would to be transported by ENaC and thus potassium secretion would not take place. A possible role of NCC after infusion with hypertonic saline is purely speculative as we did not measure the activity of NCC. Perhaps we did not see the positive effect on potassium secretion within our time limits. However, the potassium transport is complex and factors modulating potassium transport, such as altered tubular flow and aldosteron, are many.

After volume expansion with isotonic saline the oncotic pressure is slightly reduced, which leads to an immediately increase in GFR and smaller reabsorption of water in the proximal tubule. We measured a small increase in GFR and UO output. Sodium excretion increased, but u-ENaCγ, p-Na, p-osm and p-AVP remained unchanged, thus the findings were as we expected. Regarding NCC, one would not expect any change in NCC mediated sodium reabsorption during isotonic saline.

No study has ever evaluated u-ENaCγ during water diuresis. In our study, we measured a trend towards a reduction in u-ENaCγ after glucose infusion reflecting a small reduction of sodium reabsorption via ENaC in the principal cell. As previously mentioned, we measured a 2% fall in p-osmolality after glucose infusion, which theoretically should trigger a decrease in AVP. We did not detect a fall in p-AVP, presumably due to a low p-AVP caused by oral water loading in advance or the fact that the measurements of p-AVP concentration may not be sensitive enough to detect small changes. It could be hypothesized that the decrease in u-ENaCγ could be due to a lack of AVP binding to V2 receptors in the basolateral membrane of the principal cell. Lack of AVP stimuli leads to an increased endocytosis of ENaC channels from the membrane surface into recycling vesicles, there by decreasing reabsorption of sodium [54,55].

Thus, in the last post infusion period u-ENaCγ increased markedly after hypertonic saline infusion, was approximately at the same level after isotonic saline and decreased or tended to decrease in response to glucose infusion. The rise in p-osm and p-AVP was seen immediately after 3% NaCl infusion stopped. The delay and constant level of u-ENaCγ after hypertonic saline could be explained by the fact that it takes few minutes to increase trafficking of intracellular depots of ENaC channels into the apical membrane but several minutes to excrete ENaC into the urine after stimulation with AVP.

### Vasoactive hormones

In addition to AVP, the renin-angiotensin-aldosterone system (RAAS) is a key regulator of renal sodium excretion and thereby of body fluid volume. It is well known that sodium depletion activates and that chronic sodium load reduces the RAAS [56]. In vitro and in vivo studies have shown that aldosterone stimulates the mineralocorticoid receptor to an increased transcription of genes-encoding proteins involved in sodium transport i.e. ENaC and Na,K-ATPase [57].

Numerous studies of changes in blood volume have demonstrated that acute changes are associated with inverse adjustments of the renin-angiotensin-aldosterone system [21,27,51,58]. In the present study, volume expansion with 3% and 0.9% saline resulted in a similar and significant reduction in PRC, p-AngII and Aldo consistent with an increase in extracellular volume. This is in agreement with previous studies [51,58].

After glucose infusion, we measured no significant change in PRC, p-AngII or p-Aldo. This was expected, as glucose infusion does not cause any marked change in extracellular volume. Our study was not designed to allow any regulatory effects of aldosterone as the action of aldosterone occurs over hours or days. Therefor other factors must be implicated in the regulation of ENaC.

### Strengths and limitations

The major strength of this study was the design as a randomized crossover study with a homogenous group of healthy young men and women. The test conditions were very well defined regarding diet, sodium and fluid intake. Thus, the results are not confounded by different salt or water balance. This study explored only the acute effects of volume expansion. No doubt, we could have gained further information regarding the long-term effects of volume expansion and the urinary excretion of AQP2 and ENaCγ if the post infusion period had been longer. In addition, the study was not placebo-controlled, by means of infusion with a negligible amount of 0.9% saline. This could have distinguished the effects of volume expansion from the overall variability of water and salt reabsorption. In this study it was not possible to perform ANP measurements. It could have made a positive contribution to our results.

## Conclusions

In conclusion volume expansion with 3% and 0.9% saline clearly increased u-AQP2, while isotonic glucose decreased u-AQP2. Infusion of hypertonic saline increased u-ENaCγ, whereas u-ENaCγ was not significantly changed after isotonic saline and decreased or tended to decrease after glucose. Thus, the transport of water and sodium changed, both via the aquaporin 2 water channels and the epithelial sodium channels, during all three types of volume expansion in order to regulate and maintain water- and sodium homeostasis in the body. Changes in the renin-angiotensin-aldosterone system did not seem to bear a causal relationship with the changes in u-AQP2 or u-ENaCγ.

## Competing interests

The authors declare that they have no competing interests. The authors alone are responsible for the content and writing of the paper.

## Authors’ contributions

All authors have contributed to the manuscript. JMJ, FHM and EBP designed the project. JMJ and FHM performed the experiments and statistical analyzes. JMJ, FHM, JNB, SN and EBP wrote and edited the manuscript. All authors read and approved the final manuscript.

## Pre-publication history

The pre-publication history for this paper can be accessed here:

http://www.biomedcentral.com/1471-2369/14/202/prepub
